# Quality of diabetes health education provided by healthcare professionals to patients with type 1 and type 2 diabetes mellitus across different healthcare settings: a scoping review protocol

**DOI:** 10.3389/frhs.2026.1917504

**Published:** 2026-09-21

**Authors:** Nontsikelelo Qebengu, Mabina Mogale

**Affiliations:** Department of Public Health, Sefako Makgatho Health Sciences University School of Health Care Sciences, Pretoria, South Africa

**Keywords:** diabetes mellitus, diabetes self-management education and support, digital health, health education quality, healthcare professionals, low- and middle-income countries, mHealth, scoping review

## Abstract

**Background:**

Diabetes mellitus is a major global public health challenge associated with substantial morbidity, premature mortality, escalating healthcare costs, and reduced quality of life. High-quality diabetes health education, particularly through Diabetes Self-Management Education and Support (DSMES), is fundamental to effective diabetes self-management. However, considerable variability exists in the quality of education delivered by healthcare professionals, and existing evidence remains fragmented, particularly regarding educational quality, digital health integration, and implementation across diverse healthcare settings.

**Objective:**

This scoping review aims to map and synthesise existing evidence on the quality of diabetes health education delivered by HCPs' across diverse healthcare settings.

**Methods and analysis:**

This scoping review will follow the methodological framework proposed by Arksey and O'Malley, enhanced by Levac and colleagues, and refined by the Joanna Briggs Institute. Electronic databases will be systematically searched, and studies will be selected in accordance with predefined Population, Concept, and Context eligibility criteria. Two reviewers will independently screen studies, extract data using a standardised charting form guided by Donabedian's Structure–Process–Outcome framework, and report the review according to the Preferred Reporting Items for Systematic Reviews and Meta-Analyses Extension for Scoping Reviews (PRISMA-ScR).

**Results:**

This scoping review will provide a comprehensive synthesis of the evidence on the quality of diabetes health education delivered by healthcare professionals. The findings are expected to inform healthcare professional education and training, strengthen educational quality, support evidence-informed policy and practice, and guide future research to improve diabetes care across diverse healthcare settings.

**Conclusion:**

The scoping review will provide a comprehensive synthesis of evidence on the quality of diabetes health education delivered by HCPs'. The findings are expected to inform policy, strengthen professional training, and support evidence-based improvements in DSMES delivery, particularly in resource-limited and underserved settings.

## 1 Background and rationale

Diabetes mellitus is a major global public health challenge associated with substantial morbidity, premature mortality, escalating healthcare costs, and reduced quality of life. According to the International Diabetes Federation, an estimated 589 million adults were living with diabetes in 2024, and this number is projected to increase to 853 million by 2050 (1). Despite advances in prevention and treatment, many people continue to experience poor glycaemic control and diabetes-related complications, highlighting the need for effective strategies that support long-term diabetes self-management (2–6).

Diabetes health education is a fundamental component of comprehensive diabetes care because it equips people living with diabetes with the knowledge, skills, confidence, and problem-solving abilities needed to make informed decisions and manage their condition effectively (7–9).

This is primarily achieved through DSMES, an evidence-based, person-centred process that facilitates informed decision-making, strengthens self-management behaviours, supports problem-solving, and promotes active collaboration between individuals living with diabetes and HCPs' (7–11).

Evidence demonstrates that high-quality DSMES improves self-management behaviours, treatment adherence, glycaemic control, quality of life, and reduces the risk of diabetes-related complications (7–10, 12–14). Healthcare professionals (HCPs') play a pivotal role in delivering diabetes health education and supporting diabetes self-management across the continuum of care (15–21).

However, evidence consistently demonstrates considerable variability in the quality of diabetes health education delivered across healthcare settings, including differences in HCPs' diabetes-related knowledge, educational competence, communication practices, cultural responsiveness, adherence to evidence-based standards for diabetes self-management education and support, and opportunities for continuing professional development (15–24). These inconsistencies may adversely affect patient engagement, self-management behaviours, glycaemic control, and long-term health outcomes. These challenges are particularly pronounced in low- and middle-income countries (LMICs), where shortages of trained HCPs', high patient volumes, inadequate educational resources, and limited opportunities for continuing professional development constrain the delivery of structured, evidence-based diabetes health education (25–29, 39). Consequently, diabetes health education is frequently delivered inconsistently, limiting its effectiveness and contributing to inequities in diabetes care.

Alongside these challenges, the growing integration of digital health innovations such as mobile applications, telehealth platforms, and other technology-enabled approaches into diabetes health education underscores the need for HCPs' to receive adequate training and sustained support (7–9, 27–31). The quality of diabetes health education can be conceptualised using Donabedian's Structure–Process–Outcome framework, which distinguishes between structural factors, educational processes, and patient outcomes and provides a useful conceptual lens for evaluating educational quality (32).

Although the literature on diabetes health education has expanded considerably, existing evidence remains fragmented and has largely focused on the effectiveness of diabetes self-management education interventions or patient outcomes rather than comprehensively examining the quality of diabetes health education delivered by HCPs'. To the best of our knowledge, no scoping review has systematically synthesised evidence on HCPs' educational competence, quality dimensions, integration of digital health technologies, and implementation challenges across diverse healthcare settings. Addressing this gap will provide an evidence base to inform HCP training, strengthen educational quality, and support policy and practice. Despite the growing body of literature on diabetes education, existing evidence remains fragmented, and no scoping review has comprehensively mapped the quality dimensions, digital health integration, and implementation challenges associated with healthcare professional-delivered diabetes health education.

## 2 Aim and objectives

### 2.1 Aim

This scoping review aims to map and synthesise existing evidence on the quality of diabetes health education delivered by HCPs' across diverse healthcare settings.

### 2.2 Objectives

- To map the range, nature, and characteristics of the existing literature on diabetes health education delivered by HCPs'.
- To examine reported dimensions of educational quality, including HCPs' knowledge, skills, communication practices, cultural competence, and adherence to evidence-based DSMES standards.
- To explore how digital health innovations are integrated into diabetes health education by HCPs' and how this influences quality and fidelity.
- To identify gaps, challenges, and barriers to delivering high-quality diabetes health education, particularly in resource-limited and underserved settings.
- To synthesise implications for practice, policy, and training, and to identify priorities for future research.

### 2.3 Review questions

#### 2.3.1 Primary review question

What evidence exists regarding the quality of diabetes health education delivered by healthcare professionals across different healthcare settings?.

#### 2.3.2 Secondary questions

- How is educational quality conceptualised and assessed in diabetes health education delivered by HCPs'?
- What competencies and practices are associated with high-quality DSMES delivery?
- How are digital health tools incorporated into diabetes health education, and what challenges affect their implementation?
- What contextual and system-level factors influence educational quality across different settings?

### 2.4 Methods

This scoping review will be conducted using the methodological framework originally proposed by Arksey and O'Malley (33), incorporating the methodological enhancements recommended Levac et al. (34) and following the current guidance of the Joanna Briggs Institute for scoping reviews (33–35). The review will be reported in accordance with the Preferred Reporting Items for Systematic Reviews and Meta-Analyses Extension for Scoping Reviews (PRISMA-ScR) guidelines (36).

### 2.5 Eligibility criteria

The eligibility criteria will be developed using the Population-Concept-Context (PCC) framework recommended by the Joanna Briggs Institute to ensure that the review question, eligibility criteria, and search strategy are appropriately aligned with the review objectives (33, 35).

### 2.6 Population

- HCPs' involved in diabetes health education, including nurses, physicians, allied health professionals, and community health workers formally employed within the healthcare system or working under HCPs' supervision.

### 2.7 Concept

- Diabetes health education and DSMES delivered by HCPs'.
- Quality-related dimensions such as competence, communication, cultural relevance, educational approaches, and fidelity to DSMES standards.

### 2.8 Context

- All healthcare settings (primary care, hospitals, community, digital/remote settings).
- Studies conducted globally, with particular attention to LMIC context.

### 2.9 Types of sources

This scoping review will consider primary empirical studies employing quantitative, qualitative, and mixed-methods research designs. Eligible quantitative studies will include experimental, quasi-experimental, observational, and cross-sectional studies, while qualitative studies employing recognised qualitative methodologies will also be included. Mixed-methods studies will be eligible where they report findings relevant to the review objectives. Only peer-reviewed publications published in English between 2015 and 2025 will be included to capture contemporary evidence on diabetes health education and the integration of digital health technologies into HCP-led diabetes health education. Conference abstracts, editorials, commentaries, letters to the editor, dissertations, theses, study protocols, and opinion papers will be excluded.

### 2.10 Publication period

Only studies published between January 2015 and the date of the final search will be included. The 10-year timeframe was selected to ensure that the review reflects contemporary evidence on the quality of diabetes health education delivered by HCPs'. This period corresponds with important developments in evidence-based DSMES, including updates to international diabetes care guidelines, increasing emphasis on person-centred and culturally responsive care, competency-based HCP education, and the growing integration of digital health technologies into routine diabetes health education and support. Consequently, studies published before 2015 may not adequately reflect current educational practices, healthcare delivery models, or technological advances relevant to contemporary diabetes care. Although restricting the review to the past 10 years may exclude seminal studies that contributed to the historical development of diabetes health education and DSMES, this timeframe was selected to ensure that the evidence synthesised is relevant to current clinical practice, healthcare policy, and future research.

Studies will be excluded if they:

- Do not involve HCPs' as the primary providers or facilitators of diabetes health education (e.g., studies involving only peer-led, family-led, community volunteer-led, or patient-initiated education without HCPs' involvement.
- Focus exclusively on patient outcomes without describing, evaluating, or reporting the quality, content, delivery, or implementation of diabetes health education provided by HCPs'.
- Do not examine one or more dimensions of educational quality relevant to this review, including HCPs' competence, diabetes-related knowledge, communication practices, cultural responsiveness, adherence to evidence-based standards for diabetes self-management education and support, instructional delivery processes, or other structural, process, or outcome indicators of educational quality.
- Do not report primary empirical research, including conference abstracts, editorials, commentaries, letters to the editor, opinion papers, study protocols, dissertations, theses, book chapters, narrative reviews, or other non-empirical publications.
- Are published in languages other than English.
- Are published outside the predefined publication period.
- Have full-text articles that cannot be obtained after reasonable efforts

### 2.11 Search strategy

A comprehensive search strategy will be developed in consultation with an academic librarian to ensure methodological rigour, comprehensiveness, and reproducibility.

The search strategy will be conducted in accordance with the Joanna Briggs Institute methodology for scoping reviews and will be structured using the Population, Concept, and Context framework to align with the review objectives. Electronic databases to be searched will include MEDLINE (via PubMed), CINAHL, Scopus, Web of Science, and Embase. To minimise publication bias and identify additional relevant evidence, grey literature will also be searched, including reports from the World Health Organization, government publications, professional organisations, and relevant policy documents. The reference lists of all included studies and relevant review articles will be manually screened to identify further eligible studies. The search strategy will combine controlled vocabulary, including Medical Subject Headings in MEDLINE and equivalent subject headings in other databases, with free-text keywords using Boolean operators (AND, OR), truncation (*), and phrase searching, where appropriate (35, 37). Search terms will be developed around the Population, Concept, and Context framework and will include terms related to diabetes mellitus, HCPs’, diabetes health education, diabetes self-management education and support, educational quality, competence, communication, cultural competence, digital health, mobile health, telehealth, and primary healthcare. The search strategy will be adapted to the indexing terms and search syntax of each database. An example of the MEDLINE (via PubMed) search strategy is presented below. The complete search strategies for all databases, including Boolean search strings, Medical Subject Headings, keywords, and search limits, are provided in Supplementary Appendix S1.

### 2.12 Example search strategy (MEDLINE via PubMed)

(“Diabetes Mellitus”[Mesh] OR “diabetes mellitus” OR “type 1 diabetes” OR “type 2 diabetes”) AND (“Health Education”[Mesh] OR “Patient Education as Topic”[Mesh] OR “diabetes self-management education” OR “diabetes self-management education and support”) AND (“Health Personnel”[Mesh] OR “healthcare professional*” OR nurse* OR physician* OR dietitian* OR pharmacist* OR diabetes educator* OR allied health professional* OR clinician*) AND (quality OR competence OR knowledge OR communication OR “cultural competence” OR “digital health” OR telehealth OR mHealth).

### 2.13 Study selection

All records identified through the database searches will be exported to EndNote 20 for reference management and duplicate removal. Following de-duplication, the remaining records will be imported into Rayyan, a web-based systematic review management platform, to facilitate independent study screening by two reviewers (38). The study selection process will be conducted in two stages in accordance with the predefined eligibility criteria based on the PCC framework (35). In the first stage, titles and abstracts will be independently screened to identify potentially eligible studies. In the second stage, the full texts of all potentially eligible articles will be retrieved and independently assessed for inclusion by the same two reviewers (34, 35). Reasons for the exclusion of full-text articles will be documented and reported. Any disagreements between reviewers at either stage will be resolved through discussion and consensus. Where consensus cannot be reached, a third reviewer will be consulted to determine the final decision (34, 35). The study selection process will be documented and reported using the PRISMA-ScR flow diagram, which will summarise the number of records identified, screened, excluded (with reasons), and included in the final review (36). A template of the study selection process is provided in Figure 1.

**Figure 1:**
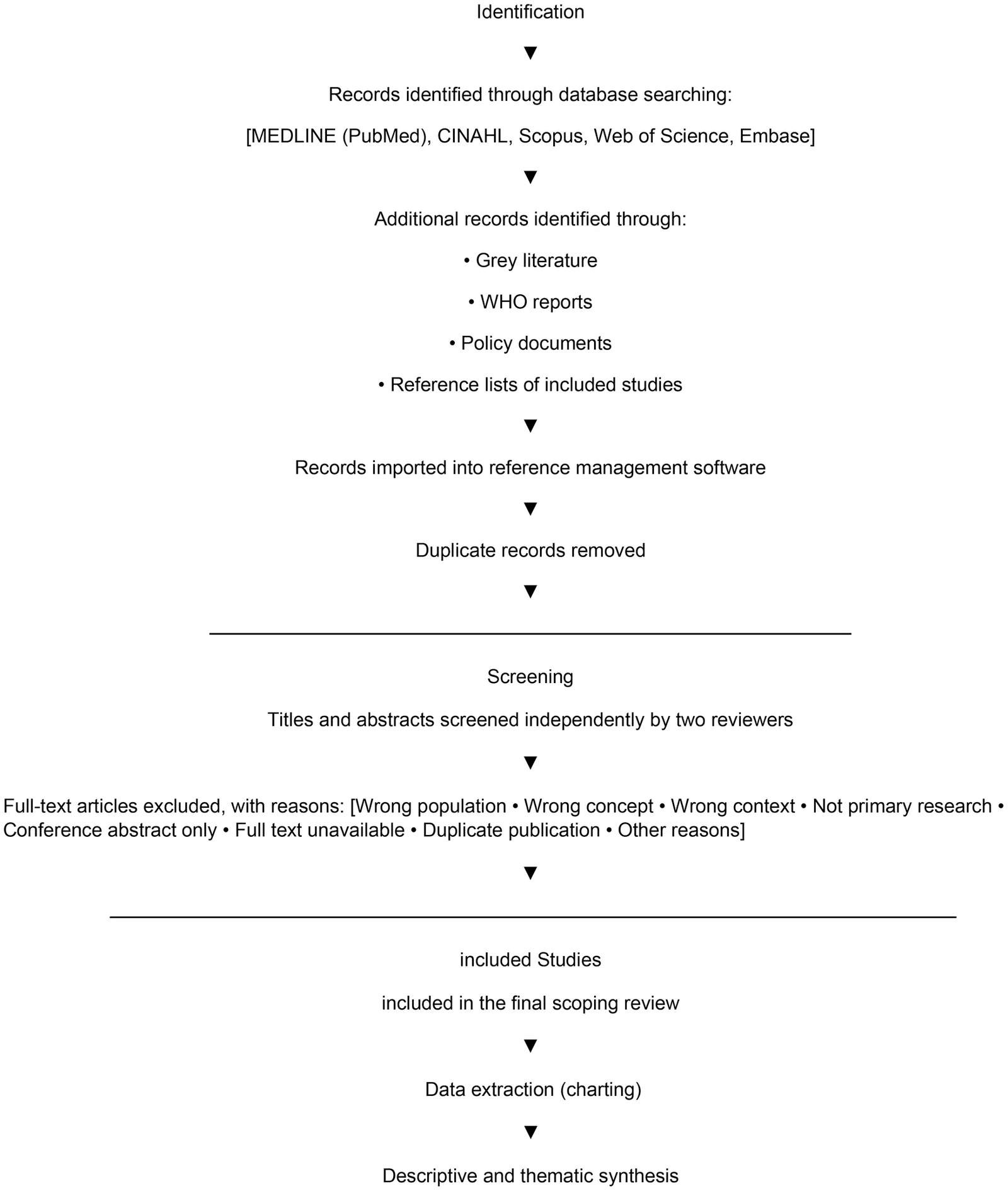
PRISMA Flow diagram.

### 2.14 Data extraction

Data extraction (data charting) will be guided by Donabedian's Structure–Process–Outcome framework, which provides the conceptual basis for defining and evaluating the quality of diabetes health education delivered by HCP's (32).

A standardised data extraction (charting) form (Supplementary Appendix S2) will be developed to systematically capture relevant information from each included study in accordance with the Joanna Briggs Institute guidance for scoping reviews (35).

The charting form will be piloted on a sample of eligible studies and refined, where necessary, to ensure clarity, consistency, and completeness of data extraction (34, 35). The extracted data will include bibliographic information, study characteristics, healthcare setting, participant characteristics, educational interventions or practices, and dimensions of educational quality. The extracted data will be organised according to the three domains of Donabedian's Structure–Process–Outcome framework (32):

- Structure: HCP training, organisational resources, educational materials, and healthcare setting.
- Process: Communication practices, cultural responsiveness, educational approaches, adherence to evidence-based standards for diabetes self-management education and support, and integration of digital health technologies.
- Outcomes: Patient knowledge, self-management behaviours, self-efficacy, glycaemic control, patient satisfaction, and quality of life.

Additional information relating to implementation barriers, facilitators, key findings, and authors' recommendations will also be extracted. Two reviewers will independently chart data from all included studies. Any discrepancies will be resolved through discussion and consensus. Where consensus cannot be reached, a third reviewer will be consulted to reach a final decision (34, 35).

The final data charting form is provided in Supplementary Appendix S2.

### 2.15 Pilot testing

The draft data extraction form will be piloted on a small sample of included studies to assess its clarity, completeness, and consistency (34, 35). Following pilot testing, the form may be refined to ensure that all relevant variables are adequately captured. Any modifications made during the review process will be documented and reported in the final scoping review.

### 2.16 Data synthesis

Data will be synthesised descriptively and thematically in accordance with the review objectives and current guidance for the conduct of scoping reviews (35). Quantitative findings will be summarised using descriptive statistics, including frequencies, counts, and percentages, to provide an overview of the characteristics of the included studies. Qualitative findings will be synthesised thematically to identify recurring themes, patterns, barriers, facilitators, and contextual factors influencing the quality of diabetes health education delivered by HCPs' (35). To enhance conceptual coherence, findings will be interpreted using Donabedian's Structure–Process–Outcome framework, enabling the identification and synthesis of Structure–Process–Outcome dimensions of educational quality across the included studies (32). Findings will be presented using summary tables, charts, figures, and narrative synthesis to map the breadth and nature of the available evidence, identify knowledge gaps, and highlight priorities for future research (33, 35). The review findings will be reported in accordance with the PRISMA-ScR guidelines (36).

### 2.17 Ethics and dissemination

Ethical approval is not required for this scoping review because it will synthesise evidence from publicly available sources and will not involve the collection of primary data from human participants (35). The findings will be disseminated through publication in a peer-reviewed journal and presentations at relevant academic, professional, and policy forums. The review will provide evidence to inform clinical practice, HCPs' education and training, policy development, and strategies to strengthen the quality and effectiveness of diabetes self-management education.

### 2.18 Expected contribution

This scoping review will provide a comprehensive synthesis of the evidence on the quality of diabetes health education delivered by HCPs' across diverse healthcare settings. The review will identify the key dimensions of educational quality and examine the factors that influence effective educational delivery. It will evaluate how digital health technologies are integrated into diabetes health education and pinpoint knowledge gaps in the existing literature. Additionally, the review will provide evidence-informed recommendations to enhance HCP training, improve the quality of diabetes health education, guide future research, and inform policies and practices aimed at bettering diabetes care.

### 2.19 Limitations of the review protocol

This scoping review has several anticipated limitations. First, only studies published in English will be included, which may exclude relevant evidence published in other languages and introduce language bias, particularly in non-English-speaking regions of LMICs (35). Second, the review will be limited to studies published between January 2015 and December 2025 to capture contemporary evidence reflecting current diabetes health education practices, evolving models of healthcare delivery, and the increasing integration of digital health technologies. Subsequently, earlier studies that contributed to the development of diabetes health education quality frameworks may not be included.

Third, the concept of educational quality is not consistently defined or operationalised across the literature. To address this challenge, Donabedian's Structure–Process–Outcome framework will be used as the conceptual framework for data extraction and synthesis (32). However, variation in definitions, outcome measures, and reporting practices may limit direct comparisons between studies. Finally, the included studies are expected to differ in their research designs, healthcare settings, healthcare professional groups, educational interventions, and study contexts. Because of these differences, the findings will be summarised descriptively rather than combined statistically. This is consistent with the purpose of a scoping review, which aims to map and describe the available evidence and identify knowledge gaps, rather than evaluate the effectiveness of interventions (33, 35).
