# Supplementary material for: Quality of diabetes health education provided by healthcare professionals to patients with type 1 and type 2 diabetes mellitus across different healthcare settings: a scoping review protocol
Source: Front Health Serv. 2026 Sep 21;6:1917504. doi: 10.3389/frhs.2026.1917504 (PMC13635125; doi:10.3389/frhs.2026.1917504)
Supplement: Supplementary file 1 [file Table1.docx]

**Supplementary Appendix 2. Draft Data Extraction (Charting) Form**

The data extraction form was developed in accordance with JBI methodology for scoping reviews. It will be piloted on a sample of eligible studies and refined where necessary to ensure that all relevant data are consistently captured. Two reviewers will independently extract data from the included studies, with any discrepancies resolved through discussion or consultation with a third reviewer.

**Table1: Data Extraction (Charting) Form**

| Section | Data Item | Description/Examples |
| --- | --- | --- |
| Bibliographic Information | Author(s) | First author and co-authors |
|  | Year of publication | Publication year |
|  | Country | Country where the study was conducted |
|  | Journal | Journal or publication source |
| Study Characteristics | Aim/objective | Purpose of the study |
|  | Study design | Qualitative, quantitative, mixed methods, systematic review, etc. |
|  | Healthcare setting | Primary healthcare, hospital, community, outpatient clinic, specialist clinic, digital/remote setting |
| Population | Healthcare professional category | Nurse, physician, diabetes educator, pharmacist, dietitian, allied health professional, community health worker, multidisciplinary team |
|  | Participant characteristics | Sample size, profession, years of experience (where reported) |
|  | Diabetes population | Type 1 diabetes, Type 2 diabetes, gestational diabetes, mixed population |
| Concept: Diabetes Health Education | Educational intervention/practice | Description of diabetes health education or DSMES delivered |
|  | Educational approach | Individual education, group education, structured DSMES, counselling, coaching, education programme, multidisciplinary education |
|  | Educational content | Lifestyle modification, medication adherence, blood glucose monitoring, nutrition, physical activity, foot care, complication prevention, psychosocial support |
| Educational Quality | Knowledge | Evidence relating to HCP knowledge of diabetes education |
|  | Competence/skills | Clinical competence, educational skills, confidence, training |
|  | Communication practices | Patient-centred communication, shared decision-making, counselling techniques |
|  | Cultural competence | Culturally appropriate education, language considerations, health literacy adaptation |
|  | Adherence to standards | Use of evidence-based guidelines or recognised DSMES standards |
| Digital Health Integration | Digital health component | mHealth, eHealth, telehealth, telemedicine, mobile applications, SMS, web-based education, electronic decision-support systems (where applicable) |
|  | Role in education | How digital health was used to support or enhance practitioner-delivered diabetes education |
| Barriers and Facilitators | Barriers | Individual, organisational, health system, resource, or contextual barriers affecting educational quality |
|  | Facilitators | Factors enabling or improving high-quality diabetes health education |
| Outcomes | Reported outcomes | Patient knowledge, self-management behaviours, glycaemic control, quality of life, patient satisfaction, HCP outcomes |
|  | Key findings | Principal findings relevant to the review objectives |
|  | Authors' conclusions | Summary of authors' conclusions |
| Notes | Comments | Additional observations relevant to synthesis |
