# Supplementary material for: Quality of diabetes health education provided by healthcare professionals to patients with type 1 and type 2 diabetes mellitus across different healthcare settings: a scoping review protocol
Source: Front Health Serv. 2026 Sep 21;6:1917504. doi: 10.3389/frhs.2026.1917504 (PMC13635125; doi:10.3389/frhs.2026.1917504)
Supplement: Supplementary file 2 [file Table2.docx]

**Supplementary Appendix 1. MEDLINE (PubMed) Search Strategy**

**Database**

MEDLINE (via PubMed)

**Search Concepts**

The search strategy was developed according to the PCC framework and combines Medical Subject Headings (MeSH) with free-text terms.

- **Population:** Healthcare professionals involved in diabetes education.
- **Concept:** Quality of diabetes health education/DSME.
- **Context:** Healthcare settings, including primary care, hospitals, community care, and digital health settings.

**PubMed Search Strategy**

("Diabetes Mellitus"[Mesh] OR diabetes mellitus[tiab] OR diabetes[tiab] OR "Type 1 Diabetes Mellitus"[Mesh] OR "Type 2 Diabetes Mellitus"[Mesh] OR "type 1 diabetes"[tiab] OR "type 2 diabetes"[tiab] OR T1DM[tiab] OR T2DM[tiab])AND ( "Health Education"[Mesh] OR "Patient Education as Topic"[Mesh] OR "Self-Management"[Mesh] OR "Diabetes Self-Management Education and Support"[tiab] OR "diabetes self-management education"[tiab]OR DSME[tiab] OR DSMES[tiab] OR "diabetes education"[tiab] OR "health education"[tiab] OR "patient education"[tiab]) AND( "Health Personnel"[Mesh] OR "Primary Health Care"[Mesh] OR healthcare professional*[tiab] OR healthcare provider*[tiab] OR health care worker*[tiab] OR nurse*[tiab] OR physician*[tiab] OR doctor*[tiab]OR clinician*[tiab] OR "diabetes educator*"[tiab] OR dietitian*[tiab] OR pharmacist*[tiab] OR "community health worker*"[tiab] OR "allied health professional*"[tiab])AND (quality[tiab] OR competence[tiab] OR competency[tiab] OR knowledge[tiab] OR skill*[tiab] OR communication[tiab] OR counselling[tiab] OR counseling[tiab] OR "cultural competence"[tiab] OR fidelity[tiab] OR adherence[tiab] OR "best practice*"[tiab]) AND("Telemedicine"[Mesh] OR "Mobile Applications"[Mesh] OR digital health[tiab] OR eHealth[tiab] OR mHealth[tiab] OR telehealth[tiab]OR telemedicine[tiab] OR "mobile application*"[tiab] OR "smartphone application*"[tiab])

**Search Limits**

- Publication period: January 2015 to the date of the final search.
- Language: English.
- Species: Humans.

**Additional Search Methods**

The reference lists of all included articles and relevant review papers will be manually screened to identify additional eligible studies. Grey literature searches will include reports from the World Health Organization (WHO), government publications, professional organisations, and other relevant policy documents. Search strategies for CINAHL, Embase, Scopus, and Web of Science will be adapted from the PubMed strategy using the appropriate database-specific controlled vocabulary (e.g., CINAHL Headings and Emtree) and search syntax.
