# Supplementary material for: Quality of diabetes health education provided by healthcare professionals to patients with type 1 and type 2 diabetes mellitus across different healthcare settings: a scoping review protocol
Source: Front Health Serv. 2026 Sep 21;6:1917504. doi: 10.3389/frhs.2026.1917504 (PMC13635125; doi:10.3389/frhs.2026.1917504)
Supplement: Supplementary file 3 [file Table3.docx]

Supplementary Appendix 3. Figure 1. Planned PRISMA-ScR Flow Diagram for Study Selection

Figure 1. Planned PRISMA-ScR Flow Diagram for Study Selection

Identification

▼

Records identified through database searching:

[MEDLINE (PubMed), CINAHL, Scopus, Web of Science, Embase]

▼

Additional records identified through:

• Grey literature

• WHO reports

• Policy documents

• Reference lists of included studies

▼

Records imported into reference management software

▼

Duplicate records removed

▼

───────────────────────────────────────────────

Screening

Titles and abstracts screened independently by two reviewers

▼

Full-text articles excluded, with reasons: [Wrong population • Wrong concept • Wrong context • Not primary research • Conference abstract only • Full text unavailable • Duplicate publication • Other reasons]

▼

──────────────────────────────────────────────────────────────

included Studies

included in the final scoping review

▼

Data extraction (charting)

▼

Descriptive and thematic synthesis

Planned PRISMA-ScR flow diagram illustrating the study identification, screening, eligibility assessment, and inclusion process for this scoping review. The completed PRISMA-ScR flow diagram, including the number of records identified, screened, excluded, assessed for eligibility, and included, together with reasons for full-text exclusion, will be reported in the final scoping review following completion of the study selection process.
